# Parametrization of energy sharing distributions in direct double photoionization of He

**DOI:** 10.1038/s41598-019-53545-z

**Published:** 2019-11-29

**Authors:** J. Andersson, S. Zagorodskikh, A. Hult Roos, O. Talaee, R. J. Squibb, D. Koulentianos, M. Wallner, V. Zhaunerchyk, R. Singh, J. H. D. Eland, J. M. Rost, R. Feifel

**Affiliations:** 1 Department of Physics, University of Gothenburg, Origovägen 6B, 412 96 Gothenburg, Sweden; 2 Department of Physics and Astronomy, Uppsala University, Box 516, SE-751 20 Uppsala, Sweden; 3 Nano and Molecular Systems Research Unit, University of Oulu, P.O. Box 3000, FI-90014 University of Oulu, Oulu, Finland; 4 Sorbonne Université, CNRS, Laboratoire de Chimie Physique-Matière et Rayonnement, F-75005 Paris, Cedex 05 France; 5 Department of Particle Physics and Astrophysics, Weizmann Institute of Science, Rehovot, 7610001 Israel; 6 Department of Chemistry, Physical and Theoretical Chemistry Laboratory, Oxford University, South Parks Road, Oxford, OX1 3QZ United Kingdom; 7 Max-Planck-Institut für Physik komplexer Systeme, Nöthnitzer Str. 38, D-01187 Dresden, Germany

**Keywords:** Atomic and molecular interactions with photons, Electronic structure of atoms and molecules

## Abstract

We present experimental results on the characteristic sharing of available excess energy, ranging from 11–221 eV, between two electrons in single-photon direct double ionization of He. An effective parametrization of the sharing distributions is presented along with an empirical model that describes the complete shape of the distribution based on a single experimentally determinable parameter. The measured total energy sharing distributions are separated into two distributions representing the shake-off and knock-out parts by simulating the sharing distribution curves expected from a pure wave collapse after a sudden removal of the primary electron. In this way, empirical knock-out distributions are extracted and both the shake-off and knock-out distributions are parametrized. These results suggest a simple method that can be applied to other atomic and molecular systems to experimentally study important aspects of the direct double ionization process.

## Introduction

**Figure 1:**
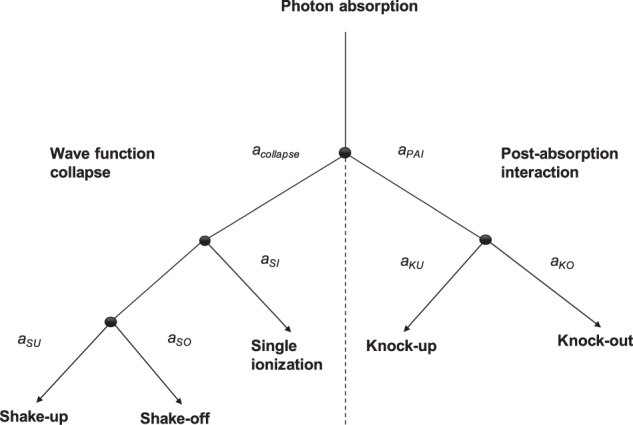
Tree diagram describing a network of sub-categorical relaxation pathways of the total transition amplitude, *a*, for a He atom that has absorbed a photon with energy above the double ionization threshold.

Single-photon direct double ionization of He has become a prototype process for investigating correlated ionization dynamics in atoms and molecules. A successful approach to modelling and conceptualizing the process was proposed and used in refs. ^1–4^, and was recently reviewed within the framework of the doctoral thesis of Jonas Andersson^5^ as summarised in what follows. Briefly, the approach relies on separating the process into two different mechanisms, both leading to double ionization. In the first mechanism, the primary electron, i.e. the one that interacts with the photon, transfers some of its energy to the secondary electron by a collision-like process. Such a process can lead to double ionization when the energy is shared in such way that both electrons receive a sufficient amount of additional kinetic energy that they can leave the system. This collision-like mechanism is called the knock-out (KO) mechanism. If the transferred energy is lower, the collision may instead lead to a singly ionized state, where the secondary electron was knocked up (KU) into an excited state. The other mechanism, called the shake-off (SO) mechanism, is based on a pure quantum mechanical process, where the primary electron leaves the system without interacting with the secondary electron as it leaves. This process is most rigorously defined in the asymptotic limit where the excess energy *E* → ∞ and the sudden approximation becomes exact. The wave function of the secondary electron corresponds to an eigenstate of the initial Hamiltonian of the neutral system and not to an eigenstate of the new, instantly changed one. This will lead the wave function of the secondary electron to collapse into one of the energetically accessible eigenstates of the new Hamiltonian. If the photon energy is higher than the double ionization potential, there will be a probability for the secondary electron to be ‘shaken off’ by a wave collapse into states with both electrons in the continuum. SO from the ground state in He would thus be characterized by the primary electron going out as a *p*-wave, as it takes the angular momentum of the absorbed photon, and the secondary electron as an *s*-wave. The collapse of the wave function may also lead the system into states where the primary electron is in the continuum and the secondary electron stays bonded to the nucleus. The secondary electron may thus be found either in the ground state of the new Hamiltonian or having been ‘shaken up’ (SU) into an excited state. Such excited states would only include excitations into *ns*-states in He^+^. An overview of the possible relaxation network is shown in Fig. 1. In addition to the KO and SO mechanisms, recent experimental studies have identified a third ionization mechanism called the *quasifree* mechanism^6,7^. The mechanism was predicted by Amusia *et al*.^8^ in 1975 and results in the two electrons being emitted back to back with similar energy. The mechanism relates to the non-dipole part of the interaction and its contribution to the direct double ionization cross section is therefore small (~1% at a photon energy of 800 eV)^6^. It is therefore assumed negligible for the energy range considered in this study. For the rest of this work, we will focus primarily on the two paths in Fig. 1 that leads to double escape. A total, fully differential transition amplitude for double escape was defined by Pattard *et al*.^2^ in terms of a sum of two independent transition amplitudes representing KO and SO,

1 $${a}_{f,i}={a}_{f,i}^{{\rm{KO}}}+{a}_{f,i}^{{\rm{SO}}}.$$

By taking the modulus squared of Eq. 1 and integrating over the angles, one gets the total energy sharing distribution, *S*_tot_ as

2 $${S}_{{\rm{tot}}}(\varepsilon ;E)={|{a}_{f,i}^{{\rm{SO}}}|}^{2}+{|{a}_{f,i}^{{\rm{KO}}}|}^{2}+{C}_{{\rm{int}}},$$

where *C*_int_ describes the interference between the two mechanisms and *ε* the kinetic energy of an electron. Based on the definition of SO and KO, proposed by Pattard *et al*.^2^, and the assumption of negligible interference (*C*_int_ = 0), as suggested for He^4^, we can write this distribution as a mixture of two independent distributions

3 $${S}_{{\rm{tot}}}(\varepsilon ;E)={S}_{{\rm{KO}}}(\varepsilon ;E)+{S}_{{\rm{SO}}}(\varepsilon ;E),$$

representing the energy sharing distribution of the KO and SO mechanism, respectively. Strong angular differences in how the two mechanisms populate the same energy eigenstates is thought to explain why the role of interference appears to be negligible, which has gained further support by angularly resolved experiments^9,10^.

The SO mechanism depends, by definition^2^, only on correlations in the initial state wave function, while the KO mechanism takes all post-absorption electron-electron interaction into account. Hence, dynamical information from the energy distribution during the post-absorption process is exclusively found in *S*_KO_. Experimental data with information reflecting the dynamics on time-scales considered in direct double photoionization is difficult to obtain. Experimental benchmarks for *S*_KO_ are thus interesting for indirect testing of the validity of different dynamical models for direct double photoionization.

## Results

By detecting the kinetic energy of both ejected electrons in a direct double ionization event, one can estimate *S*_tot_ experimentally. Having an experimental estimate of *S*_tot_ and an analytical formula for *S*_SO_ at hand, it is straightforward to extract *S*_KO_. To arrive at a formula for *S*_SO_, we rely on the same procedure as Schneider *et al*.^3,4^. We assume that one electron has been removed suddenly, and that the residual electron can be described with a hydrogenic *ns*-wavefunction, *ψ*(**r**), with effective charge *Z*_eff_. We let the wavefunction of the residual electron collapse into a free electron Coulomb wavefunction, *ν*,

4 $${a}^{{\rm{SO}}}({\bf{p}})=\int {\nu }_{{\bf{p}}}^{\ast }({\bf{r}}){\psi }_{Z{\rm{eff}}}({\bf{r}})d{\bf{r}},$$

where *ν* corresponds to an *s*-wave. From conservation of energy,

5 $$E={p}_{1}^{2}/2+{p}_{2}^{2}/2\equiv {\varepsilon }_{1}+{\varepsilon }_{2},$$

which means that the kinetic energy of either the primary, *ε*_1_ or secondary electron, *ε*_2_, immediately defines the energy of the other. As the electrons are indistinguishable, we symmetrize *a*^SO^ about *ε* = *E*/2. Integrating over the angles of the shake-off electron, the final form for the energy sharing distribution of the SO mechanism reads

6 $${S}_{{\rm{SO}}}(\varepsilon ;E,{Z}_{{\rm{eff}}})=\frac{1}{2}({|{a}^{{\rm{SO}}}(\varepsilon )|}^{2}+{|{a}^{{\rm{SO}}}(E-\varepsilon )|}^{2}).$$

**Figure 2:**
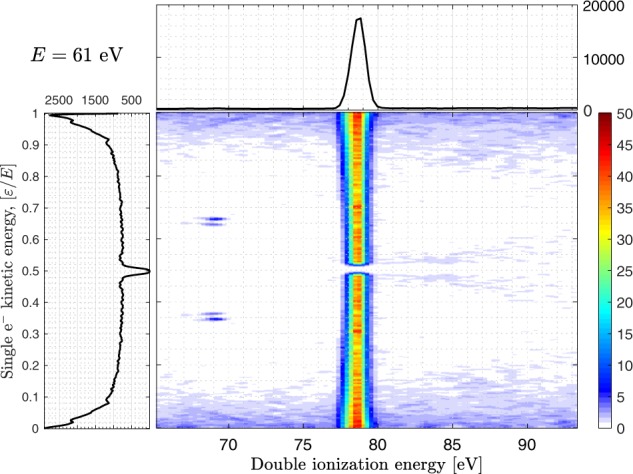
2D histogram illustrating the coincidence detection of two single electron kinetic energies and a sum corresponding to the double ionization energy of He. The photon energy was 140 eV. The map shows the continuous sharing involved in the production of single He^2+^ and the nature of the surrounding noise. The islands seen at DIP ~69 eV are Auger features from residual noble gas used for calibration, and the intensity drop at equal energy sharing is related to the dead time of the detector.

We estimated *S*_tot_ experimentally for several excess energies in the range between 11–221 eV, using a coincidence detection technique described in the methods section. The coincidence detection setup allows identification of events with a particular total kinetic energy of two electrons originating from the same ionization event. The total kinetic energy of the two electrons can be used to identify the double ionization potential (DIP)

7 $${\rm{DIP}}=h\nu -{\varepsilon }_{1}-{\varepsilon }_{2},$$

corresponding to a particular final state that the process led to. Such identification can be seen in the 2D-histogram in Fig. 2. This figure shows how a particular DIP correlates with two individual electron kinetic energies. The graph on the top of this histogram represents the projected intensity onto the *x*-axis and the graph on the left side represents the projection onto the *y*-axis. We obtain the experimental shape for *S*_tot_ by forming a graph similar to the one shown on the *y*-axis in Fig. 2 but only for the electrons involved in the formation of He^2+^. To extract good estimates of the distributions it is crucial to not only maximize the signal-to-noise level but to remove the influence of the noise profile in each bin forming *S*_tot_. To ensure this, noise estimates were subtracted for each selected DIP region. The intensity and shape of the noise was estimated by taking slices of the same size adjacent to the final state in the 2D-map. The subtracted intensity map was integrated over the final state region to form *S*_tot_. By normalizing *S*_SO_ so that

8 $${P}_{{\rm{SO}}}(E)=\frac{{\sigma }_{{\rm{SO}}}}{{\sigma }^{++}}={\int }_{0}^{E}{S}_{{\rm{SO}}}(\varepsilon ;E)\,d\varepsilon ,$$

we can extract *S*_KO_ by

9 $${S}_{{\rm{KO}}}(\varepsilon ;E)={S}_{{\rm{tot}}}(\varepsilon ;E)-{S}_{{\rm{SO}}}(\varepsilon ;E),$$

after having normalized *S*_tot_ to a proper probability distribution, such that

10 $${P}_{{\rm{tot}}}(E)={\int }_{0}^{E}{S}_{{\rm{tot}}}(\varepsilon ;E)\,d\varepsilon =1.$$

**Figure 3:**
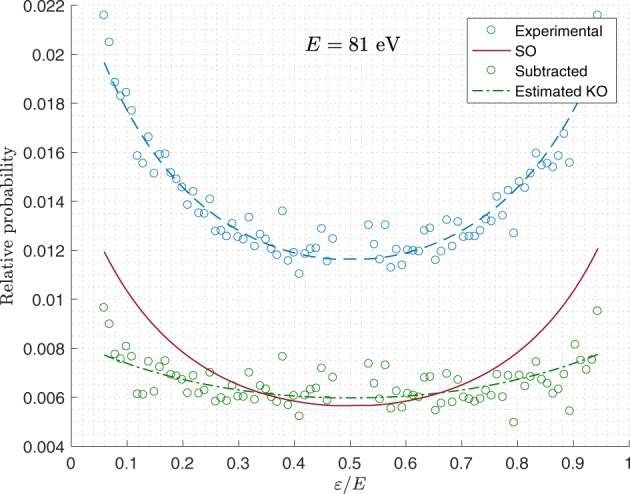
Energy sharing distributions at *E* = 81 eV. The blue dashed line shows the estimated total sharing distribution, the solid red line the calculated SO contribution and the green dot-dashed curve the KO distribution obtained by subtracting the SO part from the total experimental distribution. Raw experimental data are shown as points without any symmetrisation around *ε*/*E* = 0.5.

**Figure 4:**
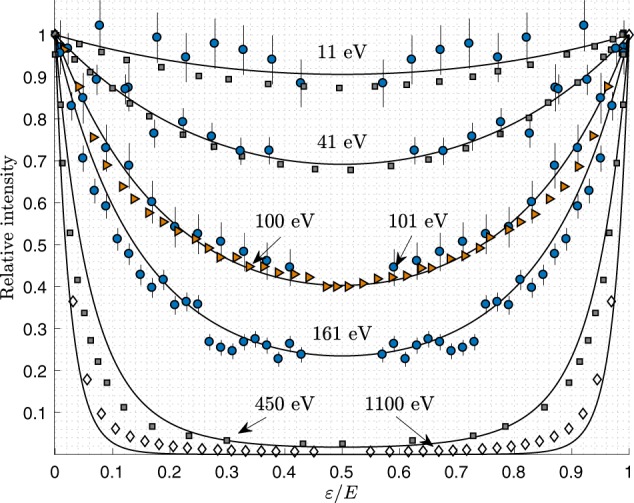
Current experimental results (solid blue circles) symmetrised about *ε*/*E* = 0.5 compared with theoretical simulations (grey squares, Schneider *et al*.^4^), experiments at *E* = 100 eV (orange triangles, Knapp *et al*.^14^, after allowing a small vertical shift), and at *E* = 1100 eV (diamonds, taken from the master thesis of Grundmann, carried out in the group of M. Schöffler and R. Dörner in Frankfurt am Main^19^). The solid black curve represents expected curve from the parametric fit presented in Table 1.

In this way, *P*_SO_(*E*) and *P*_KO_(*E*) = 1 − *P*_SO_(*E*) correspond the conditional probabilities of SO and KO given double ionization, respectively. We define *S*_SO_ according to Eq. 6 with *Z*_eff_ = *Z*_SO_ = 1.49, as suggested by Schneider *et al*.^4^. An example of how the experimental estimate of *S*_tot_ is used to extract an estimate of *S*_KO_ is shown in Fig. 3, and a sample of measured energy sharing distributions is shown and compared with previous theoretical and experimental distributions in Fig. 4.

**Figure 5:**
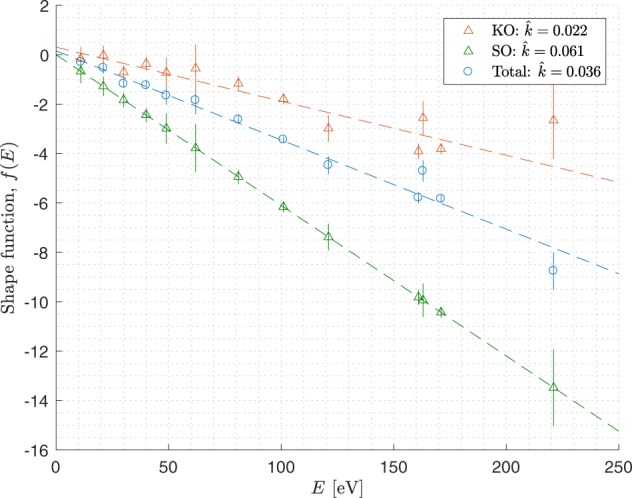
Experimentally estimated values of the shape function *f*(*E*) and linear fits through the results.

At low excess energies, the two electrons share the energy in a nearly uniform way and *S*_tot_ is nearly flat. However, at higher excess energies *S*_tot_ takes on a ∪-shaped form with edges that become sharper as the excess energy increases. This is a well known behaviour^3,4,9,11–14^ but has not been parametrized or modelled systematically over a large range of excess energies. The shape of the distributions can be well reproduced using the model function

11 $$\hat{S}\sim {e}^{f(E)\frac{\varepsilon }{E}(1-\frac{\varepsilon }{E})},$$

where *f*(*E*) is some unknown arbitrary function that sets the shape of the distribution and which may or may not depend on energy. To test *f*(*E*) we let *f* be a fit parameter for the total, the SO and the KO distributions. The plots in Fig. 5 show the optimized *f*-values for each experiment and distribution. The results indicate a linear trend with excess energy for all three distributions. This suggests that we can set *f*(*E*) = −*kE* and simplify the model to

12 $$\hat{S}\sim {e}^{-k\varepsilon (1-\frac{\varepsilon }{E})},$$

where *k* is the estimated slope of the linear trend. The model depends now on a single experimentally determinable parameter, and can be related to the differential equation

13 $$\frac{d\hat{S}}{d\varepsilon }=-\,kg(\varepsilon )\hat{S}(\varepsilon ;E),$$

where *g*(*ε*) is an odd function that ensures that the derivative changes sign at *ε* = *E*/2. The formula in Eq. 12 is obtained by solving Eq. 13 after choosing *g*(*ε*) as a first order polynomial

14 $$g(\varepsilon )=1-\frac{2\varepsilon }{E},$$

but the odd function *g*(*ε*) could in principle take on other forms.

The same fit procedure was performed for the theoretical distributions presented by Schneider *et al*.^3,4^ and the estimated fit parameters of these distributions suggest a linear trend with excess energy as well. The fitted values for the *k*-parameters are summarized in Table 1. At *ε* = *E*/2, the model reduces to an exponential function that decays as *E* increases. In principle, this means that the shapes can be readily determined simply by fitting an exponential function to the ratio between the maximum and minimum value of a distribution

15 $$R(E)=\frac{S(0;E)}{S(E/2;E)},$$

**Table 1:** Estimated values for the shape parameter *k* from simulations, *k*_theory_^3,4^ and experimental distributions, *k*_exp_, along with corresponding 95% confidence intervals, including the uncertainty in the model fitting^7^.

	Knock-out	Shake-off	Total
*k*_theory_	0.029	0.061	0.038
c.i (*k*_theory_)	(0.028, 0.030)	(0.057, 0.065)	(0.032, 0.043)
*k*_exp_	0.022	—	0.036
c.i (*k*_exp_)	(0.018, 0.026)	—	(0.033, 0.039)

for a few excess energies. It is clear from Fig. 5 that the SO, KO and total distributions all change gradually from flat to ∪-shaped as the excess energy increases. The *S*_SO_ distribution is always more ∪-shaped and sharper at the edges than the *S*_KO_ distribution.

## Discussion

Energy exchange by scattering becomes less likely when the two electrons have a large difference in their momenta. Hence, it becomes more likely to observe a secondary electron that has received just enough kinetic energy to be ionized when the primary electron is fast. This explains the ∪-formation of the KO distributions and its dependence on excess energy. The model for the SO distributions is constructed by the sum of a decaying function, centred at *ε* = 0, and the same function but reflected and centred at *ε* = *E* (see Eq. 6). The derivatives of these two partial functions, and hence the shape of the SO distribution, is defined by the effective shake charge used in Eq. 4. The photon probes the correlated two-electron wave function of the initial state and essentially filters on the primary electron positions that are most likely to absorb at a given frequency. This filtering of the initial state wave function defines the effective screening felt by the shake electron. Hence, the effective shake charge may depend on energy, and to treat it as constant may not hold true over a large range of excess energies. The significance of a varying effective shake charge is an interesting aspect but is difficult to probe with this technique, and such analysis would require clean SO-distributions recorded over a long range of photon energies.

**Figure 6:**
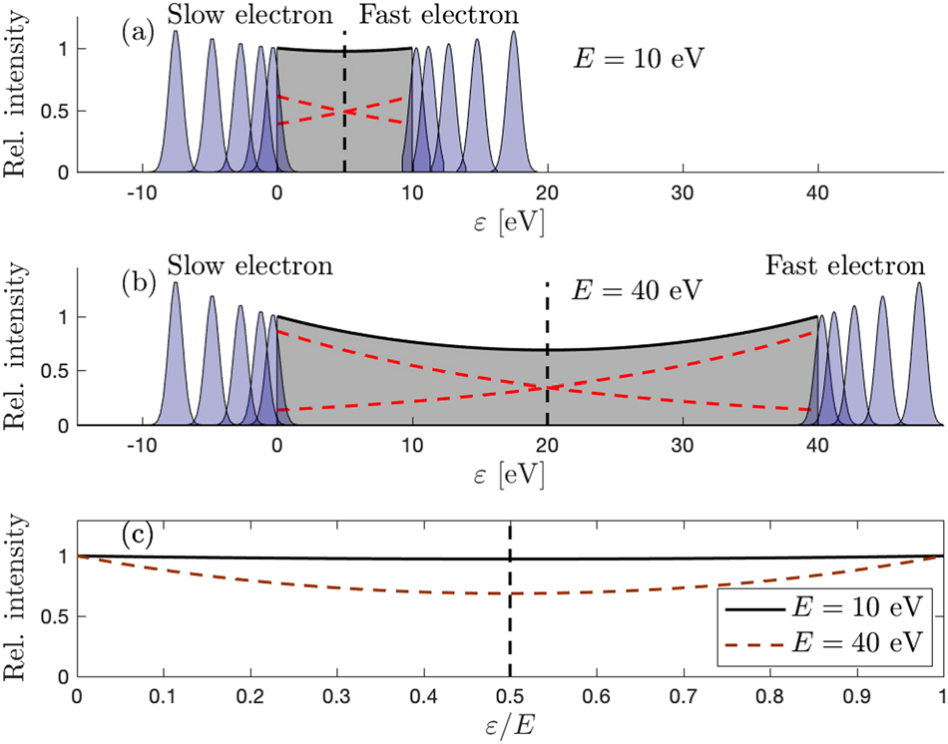
Schematic illustration of the overlap region between the partial distributions of the slow and fast electrons (red dashed curves). (**a**) Low excess energies: The overlap is strong over the entire continuum region, causing a flattening of the resulting energy sharing distribution (black curve). (**b**) High excess energies: The overlap is reduced as the distance between the maxima of the two partial distributions increases. The slopes of the red dashed curves are the same in both plots. (**c**) The relative effect of overlap for the two different excess energies.

However, the gradual ∪-shape formation of the SO distributions is still expected when assuming constant shapes for the two partial functions in Eq. 6 (i.e. keeping the effective charge fixed). The two functions will overlap more strongly at the symmetry point, *ε* = *E*/2, when the excess energy is low, and effectively flatten the SO distribution. In contrast, as the excess energy increases, the overlap at the symmetry point reduces, and a more pronounced ∪-shape is formed. The effect can be seen for the two partial functions at two arbitrary excess energies in Fig. 6a,b, and the resulting SO distributions for the two excess energies are compared in Fig. 6c.

Figure 6 also illustrates the interesting transition region where the discrete and continuum parts of the spectrum meet. As far as we know, this transition region has not yet been studied in single-photon double ionization of He, in terms of the two ionization mechanisms. Studying the transition region could potentially give further insights into the interplay between the transition channels leading to satellite states (KU and SU) and the channels leading to direct double ionization (KO and SO). Referring to the smooth merger of the Rydberg series and the continuum in single-photon absorption cross section of hydrogen, one might expect a similar merging of the SU Rydberg series and the SO continuum for high excess energies. If so, there might even be an observable enhancement of the higher *n* s-states due to a continued overlap of the two partial distributions into the discrete part of the spectrum. This is, however, beyond the scope of the present study.

## Conclusion

We have demonstrated a method to parametrize the energy sharing distribution of two electrons ionized in single-photon double ionization of He. The method relies on a single parameter that can be readily obtained by comparing the minimum and maximum value of the distribution curve. The parameter sets the shape of the distribution and how it depends on the excess energy. The complete experimental distributions were used to indirectly extract the KO distributions by using a theoretical SO model. This method requires no additional angular information for testing the difference of KO and SO. In using theoretical SO distributions, experimental KO distributions were analyzed and parametrized in a similar manner as the total sharing distributions. A parametrization and full characterization of the SO and KO mechanisms was achieved separately for excess energies between 11–221 eV. The separation revealed that there is a significant difference in how the ∪-shape evolves for the two ionization mechanisms. This way of separating the KO distribution could potentially serve as a relatively straightforward way for experimentalists to form benchmark distributions for testing dynamic correlations in single-photon double ionization events. Furthermore, this way of separating the two contributions can help in probing the importance of interference between KO and SO at intermediate excess energies. Finally, a proper energy sharing characterisation could simplify analyses of doubly ionized states, formed by a mixture of direct double ionization and single ionization followed by autoionization. A proper subtraction of the continuous direct double photoionization curve from such mixture spectra would greatly simplify the analysis of the interesting interference phenomena between discrete and continuous states in the continuum.

## Methods

The experiments were carried out at beam line U49/2-PGM-2 of the BESSY-II storage ring in Berlin, Germany. The beam line allows selecting photon energies over a large energy range (~80–2000 eV) with high precision and accuracy. The photon energy sets the excess energy as *E* = *hν* − DIP, where DIP is the double ionization potential. The storage ring was operated in single bunch mode at approximately 1.25 MHz repetition rate. To minimize the risk of detecting slow and fast electrons originating from different light pulses, the repetition rate was reduced to about 78 kHz by a synchronized mechanical chopper^15^. A repetition rate of 78 kHz corresponds to a detection window for electron flight-times up to about 12 *μ*s, which is long enough to extract even the slowest electrons. Typical count rates for these experiments were about a few hundred electron detections per second, and the accumulation time for each excess energy about one or a few hours. The magnetic bottle spectrometer that was used^16^ collects essentially all electrons emitted from an ionization event by using a strong, divergent magnetic field in the interaction volume. The magnetic field is created by a ~1 T permanent neodymium-iron magnet and the field geometry is shaped by a conical soft iron pole piece. The electrons are guided towards a 2.2 m long flight tube held at ~10^−6^ mbar pressure. The flight tube is surrounded by a solenoid producing a weak axial magnetic field that couples to the strong field. The coupling of the two magnetic fields leads to a “bottle-neck” shape of the resulting magnetic field lines. The solenoidal field guides the electrons on spiral trajectory towards a micro-channel plate detector located at the end of the tube. The collection-detection efficiency of the spectrometer is about 55%, but most importantly effectively constant over the measured range of kinetic energies as explicitly shown in the works of Hult Roos *et al*.^17,18^, which were carried out with the same spectrometer during the same experimental campaigns as the current work. This also implies that the transmission function of the spectrometer is essentially constant over the kinetic energy range of concern for the present work. Furthermore, the collection-detection efficiency mentioned implies that it is equally likely to miss an electron pair over the whole distribution and should hence not introduce any intensity biases. However, the MCP detector has a short dead time, and two electrons of the same or very nearly the same kinetic energies are thus not detectable. This can be observed as a clear intensity drop at the center of each recorded energy sharing distribution. There is a potential risk of enhanced intensity close to the drop at the center of the distribution if the two electron signals recorded in coincidence are close enough in time to effectively overlap each other. This could lead to an enhanced signal voltage, which could make it more probable to trigger and detect an electron pair of similar (but not equal) kinetic energies. If present, this effect should primarily affect the high excess energies. The inverse relationship between flight-times and kinetic energies shifts the center-point of the sharing distributions shifts toward shorter time-of-flights, which would make an overlap in the time-domain extend over a larger region in the energy domain. The overall risk of detection biases appears to be small in the current experimental data and the corresponding effect on the analysis negligible.

## Data Availability

The datasets generated during and/or analysed during the current study are available from the corresponding author on reasonable request.
